# Defining dosage

**DOI:** 10.1186/1471-227X-15-S1-A9

**Published:** 2015-06-24

**Authors:** Tommaso Pellis

**Affiliations:** 1 Department of Anesthesia and Intensive Care, Santa Maria degli Angeli Hospital, Pordenone, Italy

## 

The landmark studies on therapeutic hypothermia have demonstrated an improved outcome in comatose patients after cardiac arrest targeting a temperature of 33°C for 24 hours [1,2]. The investigated temperature of 33°C was derived from the results of animal studies suggesting the best compromise between neuroprotection and adverse events.

In the decade that followed, no randomized controlled trial of hypothermia was conducted. Thus, hypothesis-generating data from clinical registries were used to direct future investigations [3]. Surprisingly, data from a large registry of almost 1,000 patients and other observational studies were unable to demonstrate a benefit from the actual depth of temperature achieved, as well as time to hypothermia, time to target temperature, duration of hypothermia and rate of rewarming. This sets the premises and rationale of the Target Temperature Management Trial that investigated two different temperature regimes: 33°C and 36°C [4]. The study demonstrated identical long-term survival, 6 months neurological recovery and rate adverse events between the two temperature groups. This pragmatic trial enrolled a larger (939 patients) and less selected population compared with previous randomized studies. When looking at predefined subpopulations again there was benefit of one regimen over the other. Yet a numerical (nonstatistical) trend favors patients treated at 36°C. Most surprisingly, this nonstatistical advantage seems to favor patients likely to be exposed to a more severe injury, such as prolonged time from cardiac arrest to return of spontaneous circulation (median >25 minutes) or shock at admission (respectively HR 1.20 (95% CI 0.96 to 1.50) and HR 1.35 (95% CI 0.90 to 2.03)) [4]. Shock was previously considered an exclusion criterion due to alleged poor prognosis. In this subpopulation, arterial lactate levels were significantly higher in the 33°C throughout the intervention period of 36 hours ( *P*= 0.004) [5]. During the first week of ICU, the extended cardiovascular SOFA score – accounting also for need of vasopressors – was again significantly higher in the 33°C group between days 2 and 4 [5].

Based on the evidence so far available, the most recent Consensus on Science and Treatment Recommendations (Dallas 2015) from the International Liaison Committee on Resuscitation recommends selecting and maintaining a constant target temperature between 32 and 34°C [6]. Whether certain subpopulations of cardiac arrest may benefit from a lower (32 to 34°C) or higher (36°C) temperature remains unknown.

## Financial disclosure

TP has received speaker’s reimbursement from C. R. BARD.

## Declaration

This abstract and supplement was proposed, developed and commissioned by BMC Emergency Medicine and was funded by an educational grant from C. R. BARD, NJ, USA. The published abstract was independently prepared by the author. C. R. BARD had no input into the content.
